# Risk-taking in social settings: Group and peer effects^☆^

**DOI:** 10.1016/j.jebo.2013.06.010

**Published:** 2013-08

**Authors:** Spiros Bougheas, Jeroen Nieboer, Martin Sefton

**Affiliations:** School of Economics, University of Nottingham, University Park, Nottingham NG7 2RD, United Kingdom

**Keywords:** Experimental economics, Choice under risk, Advice, Social influence, Peer effects

## Abstract

We investigate experimentally the effect of consultation (unincentivized advice) on choices under risk in an incentivized investment task. We compare consultation to two benchmark treatments: one with isolated individual choices, and a second with group choice after communication. Our benchmark treatments replicate findings that groups take more risk than individuals in the investment task; content analysis of group discussions reveals that higher risk-taking in groups is positively correlated with mentions of expected value. In our consultation treatments, we find evidence of peer effects: decisions within the peer group are significantly correlated. However, average risk-taking after consultation is not significantly different from isolated individual choices. We also find that risk-taking after consultation is not affected by adding a feedback stage in which subjects see the choices of their consultation peers.

## Highlights

• We examine the role of consultation and group decision making on risk-taking.
• We replicate previous experiments showing higher risk-taking in groups relative to isolated individuals.
• We find peer effects in consultative groups: the decisions of individuals who consult with one another are correlated.
• We find that consultation does not change the average level of risk-taking relative to a treatment where individuals make decisions in isolation.
• We conclude that consultation effects alone cannot explain the higher level of risk-taking by groups relative to isolated individuals.

## 1 Introduction

The standard economic approach to the analysis of choice under risk emphasizes the role of individual risk preferences. In deciding how much to invest in a risky asset, individuals weigh up the costs and benefits referring to these preferences. By contrast, in many important real-world settings individuals do not take choices in isolation, and the social settings within which choices are made may influence behavior. For example, individual choices may be swayed by the opinions and decisions of others. In this paper we investigate how a common social setting, consultation with a group of peers, affects choices under risk in the laboratory.

There is abundant field evidence that people's choices are often influenced by their peers.^1^ While field studies can provide compelling evidence of correlated behavior within peer groups, identifying these as peer effects is complicated by confounding factors (Manski, 1993). Moreover, it is difficult to assess the influence of peer effects from field data, as naturally occurring control treatments where peer effects are absent but other variables are held constant are typically not available.

For these reasons we use a controlled laboratory experiment, described in Section 3, to investigate the effect of social settings on investment decisions over multiple periods. Our experiment has two benchmark treatments that replicate Sutter's (2009) experiment on decision-making in groups. In one treatment, decisions are made by isolated individuals without any communication with peers. In the other treatment, decisions are made by groups whose members can communicate and have to agree on a single group decision via electronic chat. In our two *consultation* treatments, subjects are also allowed to freely communicate with their peer group, as in the benchmark treatment with groups, before making a decision. However, each subject's earnings depend only on his or her own choices and not on the choices of others. We use this framework because direct communication between peers is an important feature of many settings where peers may influence one another.

Our focus on consultation contrasts with related laboratory studies of peer effects, discussed in Section 2, in which subjects are informed of each other's choices and may be influenced by these, but there is no direct communication between subjects (for example, Yechiam et al., 2008; Cooper and Rege, 2011; Lahno and Serra-Garcia, 2012). We do, however, control for the additional influence of seeing others’ choices by varying the degree of feedback we offer to subjects across the two consultation treatments. In one treatment we ensure that subjects are fully informed of the choices of others in their group while in the other treatment they do not receive such feedback.

Our experiment is also related to experiments where subjects give and take advice (also discussed in the next section). However, our framework departs from these studies in that we do not incentivize giving advice. Instead, the only motivations for our subjects to give or take advice are intrinsic motivations independent of financial consequences (as in many examples of peer advice in everyday life). Also, our subjects face the same task at the same time as their peers, whereas in other experiments on advice the experimental design induces differences between the experience and/or expertise of advice givers and takers.

In Section 4 we report our results. The benchmark treatments replicate previous findings of higher risk-taking by groups relative to isolated individuals (Sutter, 2007, 2009). Content analysis of the messages sent by group members shows that higher levels of risk taking are associated with messages referring to expected value maximization. We also find some evidence that risk-taking is higher in consultation groups where expected values are mentioned, although this effect is only marginally significant. Furthermore, we find that consultation does not increase average risk-taking beyond that observed among isolated individuals. Thus, simply providing direct communication between peers does not result in the higher risk-taking observed when decisions are made by groups. We do, however, find evidence of peer effects in our consultation treatments. Within consultation groups, variability in choices is significantly lower than the variability in choices between individuals from different groups; this result holds whether we explicitly inform subjects of their peers’ previous round choices or not. More generally, we do not find any evidence that informing subjects of the previous round choices and earnings in their peer group influences risk-taking when subjects already have the ability to consult with these peers through electronic chat.

## 2 Related literature

Compared to the long history of empirical and field studies of peer effects, the use of laboratory experiments to identify peer effects is a recent development. Experiments have shown the existence of peer effects in labor productivity experiments (Falk and Ichino, 2006; Eriksson et al., 2009; Bellemare et al., 2010), dictator games (Cason and Mui, 1998; Bicchieri and Xiao, 2009; Krupka and Weber, 2013), gift-exchange games (Thöni and Gächter, 2012; Gächter et al., 2012, 2013) and investment games (Mittone and Ploner, 2011).

Peer effects have also been shown to affect individual choice under risk. Yechiam et al. (2008) let subjects make binary choices under risk on a computer while looking at a real-time broadcast from another subject's choice screen, thus exposing subjects to each other's choices and outcomes. The authors report that mutual observation in pairs leads to higher risk-taking, but this effect is not observed when only one of the subjects in the pair observes the other. Cooper and Rege (2011) test for peer effects in a series of binary choices under risk and ambiguity, using feedback about other subjects’ choices as the channel for peer influence. They find that subjects are significantly more likely to change their response if it deviates from the majority choice of peers. Cooper and Rege also report that the peer influences of the majority opinion spills over into other gambles: if subjects observe the majority of their peers choosing the risky option in one choice, this makes them more likely to choose the risky option in other choices. Finally, the authors show that the peer effects are consistent with a model of ‘social regret’, the idea that obtaining a poor outcome from a gamble does not hurt as much if others have chosen the same gamble. Most recently, Lahno and Serra-Garcia (2012) also test for peer effects in binary lottery choices and find substantial evidence of peer effects, though responses to the decisions of peers depend strongly on whether peer decisions were voluntary or randomly imposed by the experimenter. Our work differs from these three studies in two important ways. First, the vehicle for peer effects in these three studies is the observation of others’ decisions, whereas in our experiment it is direct communication among peers. Second, whereas these three studies analyze binary lottery choices, we use a different task that is well-suited to analyzing the level of risk-taking and has been used in previous experimental studies of group decisions.

Direct communication between subjects has been investigated in a number of experimental studies. Schotter (2003) reviews experiments in which subjects receive recommendations from peers that have faced the same task. He presents evidence that advice changes behavior in ultimatum games (Schotter and Sopher, 2007), coordination games (Schotter and Sopher, 2003) and sequential guessing games (Çelen et al., 2010). The latter study also contains the striking result that subjects are more likely to follow another's recommendation rather than copy their action, although both variables have the same informational value. Chaudhuri et al. (2006) present evidence that advice leads to higher contributions and less free-riding in a public goods game. Kocher et al. (2009) find that receiving advice from peers in a beauty contest game is more effective than observational learning for improving performance. Schotter claims that advice increases efficiency or rationality because “the process of giving or receiving advice forces decision-makers to think about the problem they are facing different from the way they would do if no advice were offered” (Schotter, 2003, p. 196). The possibility of our consultation treatments having such an effect is particularly intriguing. Previous experimental results with our experimental set-up have indicated that groups take more risk than individuals, and that group communication about the higher expected earnings associated with risk-taking is an important factor behind the increased risk-taking (Sutter, 2007, 2009). Specifically, Sutter (2009) allows passive group members in one experimental treatment to send a single message to a single group decision-maker, and finds that the message type that is both most prevalent and effective urges the decision-maker to take more risk because it will result in higher expected earnings.

The advice in the studies cited above is intergenerational and incentivized. Subjects playing in period *t* give advice to subjects playing in period *t* + 1, and advisors receive an additional payoff that depends on the performance of their advisee. In our experiment, we do not incentivize giving advice. There is some experimental evidence that unincentivized peer advice affects the decisions of the advisee. Charness et al. (2010) report that that subjects perform significantly better in a probability-reasoning task after they discuss the task with fellow subjects. In an experiment with choice under ambiguity, Keck et al. (2012) present evidence that individual choices become more ambiguity-neutral after subjects discuss the experimental task in a group. Charness et al. (2013) also find that unincentivized advice from peers increases the percentage of ambiguity-neutral choices by individuals; the authors claim this is due to ambiguity-neutral subjects possessing a “persuasive edge” over others (Charness et al., 2013, p. 11). The authors also report that the peer effect on choices is stronger when subjects in a consulting pair are incentivized for each other's choices.

## 3 The experiment

Our experimental design has four treatments: one treatment where isolated individuals make choices under risk (IND), one treatment with group choices (GRP) and two treatments with individual choice after consultation (CONS and CONS + FDBK). More specifically, for our IND and GRP treatments, which we use as benchmark treatments, we replicate the design used by Sutter (2009). Like Sutter, we use the investment task introduced by Gneezy and Potters (1997). To allow for a faithful replication, we used Sutter's instructions, software, experimental parameters and incentive structure for these treatments.^2^ We also used Sutter's design as a basis for the new consultation treatments. Of course we use a different subject pool: Sutter's experiment used student subjects from the University of Jena (Germany) whereas we recruited our student subjects from the University of Nottingham (UK).

In the investment task, the decision-maker receives an endowment of 100 pence and chooses how much to invest in a risky asset. With probability 2/3 the asset bears a zero return, and the decision-maker earns that part of her endowment that was not invested. With probability 1/3 the asset returns 3.5 times the investment, and so the decision-maker earns her endowment plus 2.5 times her investment. That is, if the decision-maker invests *x* her earnings in a round are given by

$$\text{Earning}=\left\{\begin{array}{c} 100-x\;\text{with probability}\;2/3\\ 100+2.5x\;\text{with probability}\;1/3 \end{array}\right)$$

This task is repeated over nine rounds, with the asset returns determined by independent draws at the end of each round (using a computerized random number generator).

An expected earnings-maximizing (risk-neutral) decision-maker would invest the full endowment (*x* = 100), yielding expected earnings of £1.17 in every round. More generally, expected earnings are strictly increasing in *x*. We use the amount invested in the risky asset as a measure of risk-taking.

In treatment IND subjects are not allowed to communicate with each other and they do not receive feedback about others’ choices during or after the experiment. At the end of each round, subjects see a summary screen reminding them of their choice and informing them of their earnings for the round.

In treatment GRP, at the start of the experiment, groups of three subjects are randomly formed. Group composition is fixed for the whole experiment. In each round, group members can use an on-screen electronic chat to arrive at a consensus decision for the amount *x*. At any point during the chat, group members can submit a consensus decision by each entering the same amount *x* on their decision screens. If the values of *x* submitted by the three members are not the same, there is no consensus choice for the round and all group members receive nothing.^3^

As in treatment GRP, subjects in treatments CONS are randomly assigned to groups of three subjects that stay together for the whole experiment. The decision screen for this treatment also features an electronic chat between the group members, but the chat is used for consultation instead of reaching a consensus. This means that subjects in the same consultation group are not required to agree with others’ choices. We thus have an individual decision-making structure, plus consultation. At the end of the round, each subject sees a round summary screen reminding them of their own choice and informing them of their own earnings (as in IND).

In treatment CONS + FDBK, subjects are also assigned to groups of three in which they can consult others before taking a decision. At the end of each round, subjects also see a feedback screen with their own choice and earnings in the round. However, subjects in this treatment see an additional screen with feedback for the round, which informs them of the choices and earnings of the other two members in the peer group. Thus, subjects are not solely dependent on discussion during the consultation stage to learn about their peers’ choices.

### 3.1 Procedures

The experiment was carried out in the CeDEx laboratory at the University of Nottingham, in a total of 28 sessions between November 2011 and October 2012. We used ORSEE (Greiner, 2004) to recruit our subjects. Subjects were (mostly undergraduate) students from various disciplines, who had previously registered for participation in economic experiments. Altogether, 462 subjects took part in the experiment: 69 participated in treatment IND, 144 (48 groups of 3) participated in treatment GRP, 126 (42 groups of 3) participated in treatment CONS and 123 (41 groups of 3) participated in treatment CONS + FDBK.

In all treatments, subjects sit at computer terminals separated by dividers and are not allowed to communicate with one another (except through the experimental software in the relevant treatments). Subjects are given instructions (reproduced in the Appendix) that are read aloud. Subjects then make decisions over nine rounds, with the results of the lottery, their resulting round earnings, and accumulated earnings given in a feedback screen at the end of each round. Subjects in CONS + FDBK also received an additional feedback screen displaying the choices and earnings of other group members at the end of each round.

To resolve the lottery we assigned each individual/group a type at the beginning of the session, with equal numbers of subjects given each of the three possible types: 1, 2, and 3. At the end of each round subjects of one given type were successful in the lottery, depending on the realization of a computerized random number draw. In the consultation treatments all members of a consultation group had the same type, and thus either all members of a consultation group received a zero return on their individual investments, or all members received the positive return.

After the final round, subjects complete a questionnaire and are paid. Each subject is paid their full earnings for all nine rounds, plus a show-up fee of £2. Average subject earnings (including a show-up fee) were £11.71, with an average session time of 35 min.

## 4 Results

### 4.1 Average investment levels

Table 1 lists average investment in all treatments, averaged over all nine rounds and in blocks of three rounds. For comparability, we also include the averages of the original benchmark treatments (IND and GRP) as reported by Sutter (2009).

Average investment by individuals in our experiment closely mirrors the data from Sutter, whereas the average investment levels of our groups are slightly lower. Pair-wise comparisons between our IND and GRP treatments reject the null hypothesis of equal distributions, whether we focus on the average across all rounds or the average in three-round blocks. We thus replicate the results of Sutter (2009): risk-taking is significantly higher in groups than among isolated individuals.

Subjects in treatment IND choose in isolation, whereas subjects in the consultation treatments (CONS and CONS + FDBK) can communicate with peers in their consultation groups. We find that the opportunity to communicate with peers has a very weak effect on average individual risk-taking, with a (marginally) significant effect only in the last three rounds. This result holds for comparisons of IND with both treatments CONS and CONS + FDBK. In fact, when we focus on average risk-taking across the experiment, we find no evidence that risk-taking after consultation is different from choosing individually in isolation.

Investment levels in CONS and CONS + FDBK are remarkably similar: the average investment across nine rounds is nearly identical, and even the increase in the final rounds is similar. In fact, none of the three-round pair-wise comparisons between CONS and CONS + FDBK allow us to reject the null that investment is the same in both treatments (two-sided Mann–Whitney *U* tests, all *p* > 0.10). Recall that treatment CONS + FDBK differs from CONS in that it has an additional feedback screen that shows the previous round choices and earnings of the two other members of the consultation group. We thus find no evidence that showing subjects the choices of peers affects their risk-taking when subjects already have the ability to consult with peers.

### 4.2 Peer effects and within-group variability

By design, subjects in the IND treatment cannot influence one another's choices. Their decisions reflect their individual risk preferences and perceptions of the decision task. What about the consultation treatments? Here subjects are free to make the same choices they would make if they were isolated individuals, but they may be susceptible to peer effects: they could be influenced by the messages, or by the actual choices and earnings of other members of their consultation group. Since consultation does not increase the level of individual risk-taking, we now look for evidence of another type of peer effect: similarity of choices between peers.

**Fig. 1:**
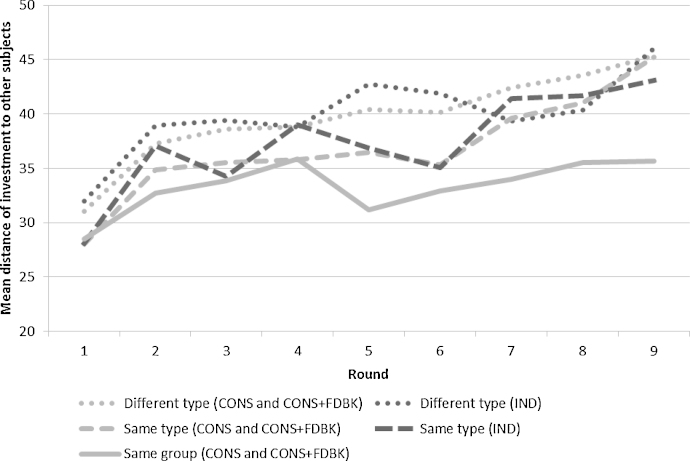
Mean distance (absolute difference) between a subject's investment and other subjects’ investment levels.

Fig. 1 shows the mean distance between a subject's investment and other subjects’ investment in each round. The ‘other subjects’ are (i) subjects of different types in the same session, (ii) subjects of the same type in a session (therefore sharing a common history of lottery wins and losses) or (iii) the two fellow consultation group members. This metric allows us to compare the consultation treatments to the individual treatment. For presentational purposes, we combine observations from both consultation treatments; using separate lines for separate consultation treatments does not affect the overall picture.

Fig. 1 shows that subjects’ investments diverge over time in both the consultation and individual treatments. However, this divergence is smaller for subjects of the same type than for subjects of different types, and even smaller for subjects in the same consultation group. The graphs in Fig. 1 suggest the existence of a particular type of peer effect: consultation with other subjects leads to decisions that are closer together than isolated individual decisions, even when taking common shocks in lottery outcomes (subjects with the same type) into account.^4^

We also analyze the similarity between decisions within consultation groups statistically. Because this type of peer effect can manifest itself across rounds (e.g. a subject copying a fellow group member's previous round investment, while said fellow group member chooses a different investment level in the current round), we look at each subject's average investment across the nine rounds. Running a simple OLS regression of investment on consultation group dummy variables, we find that group dummies are jointly significant in both consultation treatments (CONS: *F*(41, 84) = 2.63, *p* = 0.000; CONS + FDBK: *F*(40, 82) = 1.94, *p* = 0.006). The explanatory power of group dummies reflects the fact that average investment is more similar to members of the same consultation group than to those in different consultation groups.

For a non-parametric approach we compute the within-group standard deviation (WGSD) of the individual averages for each consultation group.^5^ We then take the average WGSD in our consultation treatments (19.4 in CONS; 20.8 in CONS + FDBK) and compared it to the distribution of test statistics generated using Fisher's randomization procedure.^6^ For both treatments we reject the null hypothesis that the WGSD in the consultation groups is from the same distribution as that of randomly formed three-person groups without interaction (CONS: *p* = 0.001; CONS + FDBK: *p* = 0.000). We thus find that consultation leads to significantly lower variability of investments between the three members of a consultation group, providing strong evidence that individuals do not choose independently of one another after consultation.

Finally, as a more stringent control for the possibility that intra-group correlation develops as a result of common shocks in the lottery outcomes, we repeat our analysis using choices from the first round only. Since the only difference between the consultation treatments is the feedback at the end of a round, we pool the first-round data from the two consultation treatments. Group dummies are again significant in a regression of individual investments (*F*(82, 166) = 1.31, *p* = 0.075), and the randomization test again detects significant within-group correlation (average WGSD = 22.7, *p* = 0.029). If we exclude the 47 consultation groups that do not chat in round one the effect is even stronger (*F*(35, 72) = 1.70, *p* = 0.030; average WGSD = 20.8, *p* = 0.001).

### 4.3 Communication content analysis

Communication within groups has very different effects on investment in the group versus the consultation treatments. Whereas in the group treatment the level of investment goes up relative to the individual treatment, the level of investment in consultation treatments is similar to individuals, albeit with significant peer effects in groups. To gain an understanding of why this is so, we examine the messages sent via the electronic chat communication. Two trained research assistants assigned chat messages to one or more of the following categories:

• *Amount*. A suggestion of investment amount *x* (or range of values) for the current round.
• *Cautious*. A statement that signals the individual's preference to take less risk by decreasing *x*.
• *Emotive*. A message indicating an emotional response to events in the experiment.
• *EV*. Calculations of expected value for values of *x*.
• *Off-topic*. A message that does not relate to the experimental task.
• *Risky*. A statement that signals the individual's preference to take more risk by increasing *x*.
• *Team building*. A message referring to the group itself, individual group members, or group members’ common fate.

Our research assistants received the same instructions but worked independently. Their assignments of statements to categories were cross-checked for validity by calculating Cohen's *Kappa* coefficient (Cohen, 1960) for each category. A high Kappa coefficient indicates a high proportion of agreement between the two assistants’ category judgments. Following Landis and Koch (1977), we employ a threshold Kappa value of 0.41, indicating at least moderate agreement between our research assistants. Table 2 shows the treatment-specific Kappa values for each category, as well as the average number of times a message in the category was sent in a group. We see that all of our content categories exceed the threshold Kappa value of 0.41. We also report the average number of messages in each category per group.^7^

Note that subjects send considerably fewer messages in the consultation treatments than in the GRP treatment.^8^ One plausible explanation for this difference is that groups have a clear incentive to find a consensus decision in the GRP treatment (zero earnings if consensus is not reached), whereas communication in the consultation treatments is not strictly necessary. On a related note, it could be that the higher average investment in the GRP treatment is due to the amount of communication: more chat leads to higher investments. As a simple test of this hypothesis, we calculate the correlation between the average investment and number of messages (all messages or only on-topic messages) in each group. We find no evidence of a significant correlation between these variables for any of the treatments GRP, CONS and CONS + FDBK (Spearman rank correlations, all *p* > 0.10). To investigate to what extent subjects respond to the *meaning* of chat messages, we estimate a Tobit regression where the dependent variable is the average investment in a group and with the average number of messages in each category as explanatory variables.^9^ The results are reported in Table 3.

The coefficients for message categories *Risky* and *Cautious* have the signs one would expect: *Risky* is positively correlated with investment (although not significantly in GRP or CONS), and *Cautious* is negatively correlated with investment. The coefficient on *Emotive* messages is positive and significant in the GRP treatment; one potential explanation is that groups that risk a bigger part of their endowment are more engaged in the lottery, and therefore express more emotive responses in the chat at the start of the next round. The coefficients on *EV* are positive and, except for CONS, significant: more messages referring to the expected value of various decisions are associated with higher investment. This result is in line with the hypothesis that higher investment by groups is associated with expected value maximization, as observed in the communication data presented by Sutter (2009). The fact that consultation does not lead to higher average risk-taking may be because discussion of expected value has a weaker effect than in the GRP treatment (note that the coefficient is smaller and insignificant in CONS, and although higher in CONS + FDBK the coefficient is significant only at the 10% level), or it may be simply because there is less discussion of expected value (see Table 2).

Finally, the regression results in the final column of Table 3 show no significant difference in responses to content between the CONS and CONS + FDBK treatments. This result is in line with the results on investment discussed earlier. Given that subjects have the opportunity to consult with fellow group members, showing feedback on fellow group members’ choices and earnings has no additional effect on risk-taking by individuals. We also find no evidence that subjects use the peer feedback screen in CONS + FDBK as a substitute for discussion of investment amounts in the consultation stage: the average number of messages in the ‘Amount’ category is nearly identical in both consultation treatments, even though the total number of messages sent in treatment CONS + FDBK is lower. This result may be explained by heterogeneity in susceptibility to peer effects: those who are sensitive to others’ choices will discuss choices with peers anyway, whereas more single-minded individuals will ignore others’ choices regardless of whether they learn about these through consultation or by being shown them by the experimenters.

## 5 Conclusion

Using a simple investment task we compare choices under risk by three types of decision-maker: isolated individuals, groups, and individuals who can consult each other. In line with previous research using the same investment task (Sutter, 2007, 2009), we find that groups take more risk than individuals. When individuals can consult one another we find that communication among peers leads to significant correlation of decisions within the consultation group. However, consultation has a weak effect on the *level* of risk-taking: average risk-taking after consultation is not significantly different from the average risk-taking of isolated individuals. We also find that, if subjects can already consult with others in their peer group, explicitly showing them the choices and earnings of peers does not change their behavior.

Although consulting individuals can discuss the task in the same way as group decision-makers, content analysis reveals some important differences between treatments. Perhaps most importantly, subjects in the consultation treatment exchange fewer messages than in the group treatment, including messages discussing expected values. This may explain why consultation fails to increase average investment, since mentions of expected value have a strong effect on average investment in the group treatment. These results suggest that having to make a group choice under risk is quite different from giving people the opportunity to communicate with peers.

Our consultation treatments were designed to isolate the effect of unincentivized communication between peers. If subjects had been financially motivated to provide others with investment advice – for example, if they had been paid a percentage of others’ earnings – it is possible that consultation would have had a significant effect on the level of investment. Similarly, we chose not to direct subjects to use communication in any particular way. If we had made it mandatory for subjects to justify their choice to their peers, this might have induced them to think differently about the task (and perhaps about the expected value of their choices), and may have resulted in a higher level of investment. Thus, our finding that consultation does not translate into higher levels of investment than are made by isolated individuals may reflect particular features of our design. Nevertheless, it is notable that even in our relatively simple consultation setting subjects’ decisions are influenced by their peers, as evidenced by the similarity of investment decisions within consultation groups. Further investigation of how features of the social setting influence risk-taking among peers seems a promising direction for future research.

## Figures and Tables

**Table 1:** Percentage of endowment invested.

	Sutter (2009)		Our experiment			
	IND	GRP	IND	GRP	CONS	CONS + FDBK
	(*n* = 64)	(*n* = 28)	(*n* = 69)	(*n* = 48)	(*n* = 42)	(*n* = 41)
Rounds 1–3	39.6	53.4	39.3	48.7**	38.9	40.2
Rounds 4–6	38.5	56.1	42.4	51.8*	40.5	39.0
Rounds 7–9	40.1	57.6	37.3	53.5**	44.8*	45.4*
All rounds	39.4	55.7	39.7	51.3**	41.4	41.6

The first two data columns contain the averages from the original benchmark treatments in Sutter (2009); the last four columns are the investment averages from our experiment. For IND the unit of observation is the individual. For GRP we take the consensus decision of all group members as the unit of observation. For the consultation treatments we take the average choice of the three group members as the unit of observation. The number of independent observations is indicated below the treatment names.

* Denote significant differences from our IND treatment at the 10% level, based on two-sided Mann–Whitney *U* tests.

** Denote significant differences from our IND treatment at the 5% level, based on two-sided Mann–Whitney *U* tests.

**Table 2:** Kappa values and average frequency (per group) for chat message categories.

Category	Description	Cohen's Kappa			Category frequency		
		GRP	CONS	CONS + FDBK	GRP	CONS	CONS + FDBK
Amount	Proposal of a specific amount	0.857	0.926	0.905	17.1 (10.6%)	3.1 (6.3%)	2.8 (7.6%)
Cautious	Appeal to take less risk	0.695	0.903	0.651	2.8 (1.7%)	0.8 (1.6%)	0.3 (0.8%)
Emotive	Emotive response	0.859	0.938	0.859	14.0 (8.7%)	3.8 (7.7%)	2.8 (7.6%)
EV	Expected value	0.703	0.759	0.820	2.0 (1.2%)	0.3 (0.6%)	0.3 (0.8%)
Off-topic	Off-topic	0.898	0.904	0.847	6.9 (4.3%)	5.3 (11.1%)	2.9 (8.0%)
Risky	Appeal to take more risk	0.584	0.885	0.721	3.2 (2.0%)	0.8 (1.6%)	0.4 (1.1%)
Teambuilding	Reference to group identity	0.658	0.877	0.825	5.0 (3.1%)	3.6 (7.3%)	1.8 (4.9%)

Average number of messages sent per group					161.0	49.6	36.7
Average number of classified messages					42.6	17.7	11.4

**Table 3:** Tobit regressions of avg. investment on number of messages in content categories.

	GRP	CONS	CONS + FDBK	CONS and CONS + FDBK
Amount	0.922	1.560	0.112	1.455
	(1.046)	(0.934)	(0.665)	(0.906)
Cautious	−4.801***	−5.660*	−5.650**	−5.794**
	(1.768)	(2.921)	(2.687)	(2.852)
Emotive	0.814*	1.020	0.624	0.973
	(0.484)	(0.803)	(0.474)	(0.783)
EV	4.164***	2.004	6.183*	2.217
	(1.468)	(1.870)	(3.438)	(1813)
Risky	0.617	0.843	6.722*	0.855
	(1.402)	(2.455)	(3.423)	(2.399)
Off-topic	0.443	0.0112	−0.680	0.003
	(0.265)	(0.152)	(0.436)	(0.149)
Teambuilding	−0.739	−0.330	−1.330	−0.441
	(0.733)	(0.713)	(1.143)	(0.686)
CONS + FDBK × amount				−1.288
				(1.132)
CONS + FDBK × cautious				0.216
				(3.982)
CONS + FDBK × emotive				−0.333
				(0.923)
CONS + FDBK × EV				4.195
				(4.004)
CONS + FDBK × risky				6.174
				(4.271)
CONS + FDBK × off-topic				−0.700
				(0.476)
CONS + FDBK × teambuilding				−0.661
				(1.325)

Number of observations	48	42	41	83
Prob. *>* *χ*^2^	0.00123	0.0491	0.0303	0.0117

Standard errors in parentheses. For treatment GRP we take the consensus decision of all group members as the unit of observation. For the consultation treatments we take the average choice of the three group members as the unit of observation.

* Denote significance at the 10%, level.

** Denote significance at the 5% level.

*** Denote significance at the 1% level.

## Appendix A Experimental instructions

### A.1 Treatment IND

This experiment consists of 9 rounds. In each round you will receive an endowment of 100 pence (1 pound). You must decide which part of this endowment (between 0 pence and 100 pence) you wish to invest in a lottery. The investment will be denoted as amount *X*.

The outcome of the lottery is as follows:

• With probability 2/3 (66.67%) you lose the amount *X* you have invested and your pay-off in the respective round is Pay-off = 100 − *X* pence.
• With probability 1/3 (33.33%) you win two and a half times the amount *X* you have invested in addition to your initial endowment and your pay-off in the respective round is Pay-off = 100 + 2.5*X* pence.

The actual outcome of the lottery depends on a randomly drawn number out of the uniformly distributed interval [0, 3] and on your type. There are three possible types: types 1, 2, and 3. In the first round, you will be informed about your type, which remains fixed for all nine rounds.

• Type 1 wins if the random number in a given round is from the interval [0, 1].
• Type 2 wins if the random number in a given round is from the interval (1, 2].
• Type 3 wins if the random number in a given round is from the interval (2, 3].

The random number in a given round is identical for all participants in the experiment and it will be independently drawn anew in each consecutive round. After all individuals have entered their decision, you will be informed about the outcome of the random number draw, about whether you have won or lost in the respective round, about your round pay-off and your accumulated pay-off in the whole experiment. For your final earnings, we will add up your pay-offs in all nine rounds.

In each round, you have 3 min to submit your decision. Please do not communicate with other subjects at any point during the experiment. Anybody found in breach of this rule will be dismissed without payment.

### A.2 Treatment GRP

At the start of this experiment, you will be randomly matched with two other individuals in the room to form a team of three. Team members will remain anonymous; no-one will find out who their fellow team members are during or after the experiment.

This experiment consists of 9 rounds. In each round your team will receive an endowment of 100 pence (1 pound). Your team must decide which part of this endowment (between 0 pence and 100 pence) you wish to invest in a lottery. The investment will be denoted as amount *X*. Within your team, you have to agree on a single choice of the amount *X*.

The outcome of the lottery is as follows:

• With probability 2/3 (66.67%) you lose the amount *X* you have invested and your pay-off in the respective round is Pay-off = 100 − *X* pence.
• With probability 1/3 (33.33%) you win two and a half times the amount *X* you have invested in addition to your initial endowment and your pay-off in the respective round is Pay-off = 100 + 2.5*X* pence.

The actual outcome of the lottery depends on a randomly drawn number out of the uniformly distributed interval [0, 3] and on your type. There are three possible types: types 1, 2, and 3. In the first round, you will be informed about your type, which remains fixed for all nine rounds.

• Type 1 wins if the random number in a given round is from the interval [0, 1].
• Type 2 wins if the random number in a given round is from the interval (1, 2].
• Type 3 wins if the random number in a given round is from the interval (2, 3].

The random number in a given round is identical for all participants in the experiment and it will be independently drawn anew in each consecutive round. After all teams have entered their decision, you will be informed about the outcome of the random number draw, about whether you have won or lost in the respective round, about your round payoff and your accumulated payoffs up to and including that round. For your final earnings, we will add up your payoffs in all nine rounds. Please note that each single member of a team will be paid the full earnings, which, of course, are identical for all team members.

Within your team, you and the other members have to agree on the amount *X* in each round. In order to reach agreement, you can communicate with the two other subjects via an electronic chat which will appear on your computer screen. If you have agreed on an amount *X*, please enter the amount on your input screen and confirm your entry. If the three members of your team do not enter the same amount *X* then all team members will earn zero in this round.

It is forbidden to send any message that might reveal your identity to the other team members. If you violate this rule you will not receive any payment.

### A.3 Treatments CONS and CONS + FDBK

At the start of this experiment, you will be randomly matched with two other individuals in the room to form a group of three. Group members will remain anonymous no-one will find out who their fellow group members are during or after the experiment.

This experiment consists of nine rounds. In each round you will receive an endowment of 100 pence (1 pound). You must decide which part of this endowment (between 0 pence and 100 pence) you wish to invest in a lottery. The investment will be denoted as amount *X*.

The outcome of the lottery is as follows:

• With probability 2/3 (66.67%) you lose the amount *X* you have invested and your pay-off in the respective round is Pay-off = 100 − *X* pence.
• With probability 1/3 (33.33%) you win two and a half times the amount *X* you have invested in addition to your initial endowment and your pay-off in the respective round is Pay-off = 100 + 2.5*X* pence.

The actual outcome of the lottery depends on a randomly drawn number out of the uniformly distributed interval [0, 3] and on your type. There are three possible types: types 1, 2, and 3. In the first round, you will be informed about your type, which remains fixed for all nine rounds.

• Type 1 wins if the random number in a given round is from the interval [0, 1].
• Type 2 wins if the random number in a given round is from the interval (1, 2].
• Type 3 wins if the random number in a given round is from the interval (2, 3].

The random number in a given round is identical for all participants in the experiment and it will be independently drawn anew in each consecutive round. After all individuals have entered their decision, you will be informed about the outcome of the random number draw, about whether you have won or lost in the respective round, about your round payoff and your accumulated payoffs up to and including that round. [CONS + FDBK only: You will also see a summary screen that shows the earnings of the other members of your group in the current round.] For your final earnings, we will add up your payoffs in all nine rounds.

Within your group, each individual member can choose a different amount *X* in each round. Your earnings do not depend on the choices of the other group members. Before you enter your amount *X*, you can communicate with the two other subjects via an electronic chat which will appear on your computer screen. You are free to consult with them and discuss any aspect of the experiment. However, it is forbidden to send any message that might reveal your identity to the other group members. If you violate this rule you will not receive any payment.

## Footnotes

1 Peer effects have been found in a number of settings with choices under risk or uncertainty, such as investment decisions (Kelly and O’Grada, 2000; Hong et al., 2004; Brown et al., 2008; Bursztyn et al., 2012), entrepreneurship decisions (Nanda and Sørensen, 2010; Lerner and Malmendier, 2011; Falck et al., 2012), credit-funded consumption decisions (Sotiropoulos and D’astous, 2012), criminal activity (Fergusson et al., 2002; Bayer et al., 2009), and drug and alcohol use (Fergusson et al., 2002; Duncan et al., 2005; Powell et al., 2005; Lundborg, 2006; Clark and Lohéac, 2007). For a broad review of social influence mechanisms, see Cialdini and Goldstein (2004).

2 Instructions were taken from the English translations provided in Sutter (2009). The software was a set of a z-Tree (Fischbacher, 2007) treatment files, downloaded from the journal website at http://www.e-aer.org/data/dec09/20080341_data.zip and translated to English. There is one technical difference between Sutter's original implementation and ours: whereas Sutter's ‘mixed’ group treatment uses separate chat software for group communication, we use the built-in electronic chat function of z-Tree (version 3.3.8). [0]Regarding incentives, we replace the €-sign with a £-sign for our two payment variables: the show-up fee (€2 → £2) and round endowment (€1 → £1). This means that incentives in our experiment are higher in real terms. Using the Economist's ‘Big Mac index’ (http://www.bigmacindex.org) as a proxy for PPP, we estimate that the purchasing power of £1 in 2012 is 25% higher than €1 in 2008.

3 The same tie-breaking rule was used in Sutter (2009), and it is very effective in motivating group members to reach consensus. Only 6 out of 378 decisions in our treatment GRP failed to produce a consensus decision, and never more than once per group. In each of these 6 cases, two group members agreed on the group choice but the third member submitted a different value. We use the majority choice as the data point in these cases, noting that our results are not affected by excluding these observations.

4 Recall, in order to enhance comparability between our consultation and group treatments all consultation group members were of the same type and so the return on investment was either zero for all members of a consultation group or positive for all members.

5 That is for each individual we took their average investment across all nine rounds before computing the standard deviation of the averages for that group. This measure gives a WGSD between zero and an upper bound of approximately 57.7.

6 We drew 100,000 samples of individual averages from the empirical distribution, randomly assigning individuals to groups and counting the proportion of statistics exceeding the observed statistic. More details on the properties of this statistical procedure, as well as comparisons to commonly used parametric and non-parametric techniques can be found in Moir (1998).

7 A chat message belongs to a category if it was coded as such by at least one of our research assistants.

8 Note also that fewer messages are sent in the CONS + FDBK treatment than the CONS treatment. A possible explanation for this may be that the additional feedback on other members’ choices in CONS + FDBK substitutes for communication about previous choices.

9 The regression models were also estimated with dummies for group composition demographics (age, gender, and number of economics/business students). These variables were always insignificant and do not affect our results.
